# Managing Dual Diagnosis Patients and the Importance of Assessing Capacity: Data from London Inpatient Services

**DOI:** 10.3390/brainsci15121259

**Published:** 2025-11-24

**Authors:** Iain Hyndman, Angelo Ricciardi, Olesya Ajnakina, Christian Lowe, Cemile Kalkan, Sofia Mota, Christos Theleritis, Stefano Ferracuti, Stefania Bonaccorso, Fabrizio Schifano

**Affiliations:** 1 North London NHS Foundation Trust, St Pancras Hospital, Pancras Road, London NW1 0PE, UKricciardi.angelo@gmail.com (A.R.); cemile.kalkan1@nhs.net (C.K.); 2 Department of Mental Health, ASL Roma 1, CMHT Via Monte Tomatico 9, 00193 Rome, Italy; 3 Department of Behavioural Science and Health, Institute of Epidemiology and Health Care, University College London, 1-19 Torrington Place, London WC1E 7HB, UK; olesya.ajnakina@gmail.com; 4 Institute of Psychiatry, Psychology and Neuroscience, PO20, 16 De Crespigny Park, London SE5 8AF, UK; 5 University College London Hospitals NHS Foundation Trust, 250 Euston Road, London NW1 2PG, UK; c.lowe11@nhs.net; 6 West London, Heart of Hounslow, 92 Bath Road, Hounslow TW3 3EL, UK; sofia.mota1@nhs.net; 7 2nd Psychiatry Department, Attikon University Hospital, National and Kapodistrian University of Athens, 115 28 Athens, Greece; ctheleritis@gmail.com; 8 Department of Human Neurosciences, Sapienza University of Rome, 00185 Rome, Italy; stefano.ferracuti@uniroma1.it; 9 Psychopharmacology, Drug Misuse and Novel Psychoactive Substances Research Unit, University of Hertfordshire Medical School, College Lane Campus, Hatfield AL10 9AB, UK; f.schifano@herts.ac.uk

**Keywords:** mental capacity assessment, dual-diagnosis, psychiatric inpatients, informed consent, psychotic disorders, schizophrenia, affective psychosis

## Abstract

Objective: Substance use in patients with mental disorders is often associated with worse outcomes, increased risks, and impaired decision-making. Therefore, the evaluation of mental capacity in patients with coexisting mental illness and substance use disorder (dual diagnosis) is necessary to improve clinical outcomes and mitigate risks to self and others. Design: A retrospective inspection of electronic records for patients admitted between March 2017 and August 2020 in two London inpatient facilities was conducted. Capacity was assessed using the principles set out in the Mental Capacity Act 2005. Results: A capacity assessment was recorded in 34.9% of admissions. Only 6.2% of admissions whose primary diagnosis was mental and behavioral changes due to the use of substances had a recorded mental capacity assessment. Capacity to understand the negative impact of substances was assessed in 2.1% of total admissions. Conclusions: This study indicates very low rates of mental capacity assessment across acute psychiatric admissions, with very few relating to capacity to understand the risks associated with using substances. Further research on the capacity of patients with dual diagnosis is needed. This may help to manage certain risks in this patient population.

## 1. Introduction

Dual diagnosis (DD) is defined as a coexisting severe mental illness and substance misuse [1]. Severity of psychopathology is a variable that may influence mental capacity in informed consent evaluations [2,3]. People with DD have poorer health and social outcomes (e.g., more severe psychotic symptoms, reduced treatment compliance, suboptimal physical health, and homelessness), necessitating rigorous risk assessment [4].

Substance misuse can result in significant functional impairment and disability [5]. Co-morbid substance misuse may also be a contributing factor to the increased homicide rate [6]. Between 2006–2007 and between 2015 and 2016, 48% of homicides in Scotland were committed under the influence of drugs or alcohol [6,7]. A cross-sectional study conducted in the United States found that 41.7% of people who use drugs in rural communities had been incarcerated in the preceding six months [8]. Patients with DD are more at risk of death by suicide [9,10]. More than half of the psychiatric inpatient population have DD [11,12], and half of the suicides amongst this population is committed on agreed leave. A thorough risk assessment of inpatients going on agreed leave or being discharged is therefore essential, particularly in the presence of other risk factors for completed suicide. Individuals with substance use disorders (SUDs) have shown dysfunctional decision-making processes linked to primary (e.g., acute intoxication, delirium, psychosis) and secondary (e.g., hepatic encephalopathy) conditions; the effects on capacity can be temporary or permanent and may affect the ability to understand the risks associated with substance use [13], playing a potential role in maintaining addiction behaviors [14].

Assessing capacity, specifically regarding substance use, is therefore essential to managing risk in DD patients. Clinicians are often unclear about which tool to use to assess capacity in DD patients. In England and Wales, the capacity assessment is performed by a combination of clinical assessment and the use of a two-stage legal framework established by the Mental Capacity Act 2005 (MCA) [15] for the assessment of “Impairment” and “Decision-making”. The MCA was introduced in England and Wales to provide a legal framework for decision-making assessment based on the following four components: understanding, retention, reasoning, and communication of choice. Patients who demonstrate capability in all four, with regard to the specific decision in question, are considered to have capacity and hence are able to make decisions regarding their health. Some other countries, such as Ireland [16] and Singapore [17], have similar legal frameworks for assessing capacity. Studies have shown that most psychiatric inpatients have capacity to make key decisions about treatment [18]. When supporting patients who have capacity, a patient-centered care approach which encourages shared decision-making is essential for the delivery of mental health and substance misuse treatments [19]. DD patients are often considered to have capacity to make decisions about their substance or alcohol use until proven otherwise, but a formal capacity assessment may not be documented. Capacity assessment is essential in empowering patients to make decisions about their health. In DD patients, capacity assessment can protect individuals by assessing their understanding of the risks associated with substance and alcohol misuse. However, there is generally limited literature available relating to capacity assessments in DD patients, either for diagnosis and substance use, and it is unclear how often this is assessed in clinical practice.

### Aims

This study aims to investigate how often capacity to understand the negative impact of illicit substances and alcohol is assessed in DD patients.

## 2. Patients and Methods

Electronic records related to all the patients admitted to two inpatient facilities at Highgate Mental Health Centre, North London NHS Foundation Trust (formerly Camden & Islington NHS Foundation Trust), between March 2017 and August 2020 were inspected retrospectively. The electronic clinical records are the primary clinical records system within the North London NHS Foundation Trust. This allows to search for all clinical information, including correspondence, discharge letters, capacity assessment, and events recorded throughout the patient journey. Data were anonymized for research purposes. One facility is an acute treatment 16-bed mixed ward, whilst the second is a male-only 12-bed psychiatric intensive care ward (PICU). The patients’ records were investigated in each facility by a different group of physicians. Capacity was assessed for each of the patients using the MCA [20], designed to assess people who may lack the mental capacity to make decisions about a number of decisions, including care, treatment, finances, or end-of-life decisions. The results of MCA were retrieved by inspecting a designated tab on the RIO system, which is the electronic patients record used by the Trust. Ethnicity was recorded as self-defined by patients.

### 2.1. Ethics

We completed the NHS Health Research Authority Tool as requested by the Research & Development office of the Trust. The NHS Health Research Authority Tool is a tool that helps in deciding whether a study is a research study as defined by the UK Policy Framework for Health and Social Care Research. It was concluded that our project did not require ethical approval following negative answers to the following questions: (a) “are the participants in your study randomized to different groups?”; (b) “does your study protocol demand changing treatment/patient care from accepted standards for any of the patients involved?”; and (c) “are your findings going to be generalizable?”.

### 2.2. Statistical Analyses

We described the outcomes using frequencies and percentages for categorical variables and means and standard deviations for continuous variables. Comparisons between groups of categorical variables were conducted using the chi-squared test. For count data, the rank chi-squared test was applied. Group comparisons for continuous variables were performed using the independent samples *t*-test when the data were normally distributed, or the Wilcoxon rank-sum test when non-parametric methods were appropriate. The choice between parametric and non-parametric tests was informed by an assessment of the distribution of each continuous variable. Finally, a binary logistic regression model was fitted to examine the association between diagnostic category, age, gender, and ethnicity with the likelihood of a completed capacity assessment. The model included diagnostic group (reference: other disorders), admission age, gender (reference: men), and ethnicity (reference: Asian). All results are presented as complete cases, implying that missing data were not accounted for by means of imputation. Therefore, the raw numbers across some variables may not add up to the total sample present in the data for the analyses. All analyses were conducted in R. Studio version 4.0.2 [21].

## 3. Results

Results include data from 696 admission episodes involving n = 410 patients. Table 1 outlines the demographic characteristics of those included. Out of all admissions, n = 453 (65.1%) received a capacity assessment and n = 243 (34.9%) did not. Mean age was similar between groups (43.9 vs. 42.5 years; t = −1.8, *p* = 0.067). Significant differences emerged for gender (χ^2^ = 55, *p* < 0.001), ethnicity (χ^2^ = 63, *p* < 0.001), employment (χ^2^ = 143, *p* < 0.001), and relationship status (χ^2^ = 5.5, *p* = 0.019). The majority of those assessed were male (97.1%), with a smaller majority of those not assessed also male (74.3%). This is likely explained by the inclusion of one male-only ward. Black individuals were overrepresented among assessed cases (59.3% vs. 30.6%), whereas White individuals were less represented (21.8% vs. 48.8%). Most patients were single (93.5%), particularly among assessed cases (96.7%).

Table 2 describes the clinical features of those patients who did and did not undergo a capacity assessment. A capacity assessment was documented in only 243 admission episodes (34.9%). Most assessed admissions occurred in the PICU (88.5%), whereas most non-assessed admissions were in the acute treatment ward (58.5%) (χ^2^ = 141, df = 1, *p* < 0.001). Significant differences were also observed in primary diagnoses (χ^2^ = 61, df = 5, *p* < 0.001). Schizophrenia spectrum disorders were the most prevalent among those assessed for capacity (81.8%) compared with those not assessed (60.1%). In contrast, mood (affective) disorders were more frequently recorded among non-assessed admissions (20.2%) than assessed ones (2.5%). Differences were also evident for secondary diagnoses (χ^2^ = 93, df = 14, *p* < 0.001). Secondary schizophrenia spectrum disorders were more frequent among the assessed group (14.6%) compared with the non-assessed group (7.5%). Substance-related disorders were more prevalent as secondary diagnoses in the non-assessed group (25.3%) than in the assessed group (14.6%). Finally, a higher proportion of those assessed for capacity were detained under the MHA (89.7%) compared with those not assessed (78.1%) (χ^2^ = 14, df = 1, *p* < 0.001).

Of the 243 assessments, 141 (58.3%) were of capacity to consent to medication. A total of 83 (34.2%) was related to capacity to consent to a treatment plan. In only five (2.1%) cases was capacity to understand the negative effects of substances assessed.

The logistic regression model identified several significant predictors of capacity assessment completion. Those with *mental and behavioural disorders due to substances* had significantly higher odds of a completed capacity assessment (β = 1.54, 95%CI = 0.25–3.00, *p* = 0.026). Similarly, a diagnosis of *personality disorder* was also associated with assessment completion (β = 2.05, 95%CI = 0.83–3.46, *p* = 0.002). All other diagnostic categories were not statistically significantly associated with being assessed. Moreover, older age was associated with a slightly greater likelihood of capacity assessment completion (β = 0.019, 95%CI = 0.0003–0.037, *p* = 0.047). Gender remained a strong predictor, with women significantly less likely than men to have had a capacity assessment completed (β = −2.44, 95%CI = −3.35–1.69, *p* < 0.001). Ethnicity was also significantly associated with assessment status. Compared with Asian individuals, those identifying as *Black* (β = 1.03, 95%CI = 0.33–1.78, *p* = 0.005) or *Mixed ethnicity* (β = 0.94, 95%CI = 0.046–1.86, *p* = 0.042) were more likely to have undergone a capacity assessment. No significant differences were observed for individuals identifying as *White* (*p* = 0.39) or *Other* ethnicities (*p* = 0.68).

## 4. Discussion

Our results show that a capacity assessment was conducted in only 34.9% of cases, and only 6.2% of people with mental and behavioral changes due to the use of substances had their capacity assessed. Only 2.1% of total admissions had capacity to understand the negative impact of substances evaluated. Further, diagnostic category, age, gender, and ethnicity were significant predictors of capacity assessment completion.

Although most professionals self-report high levels of confidence in their mental capacity assessment skills [22], assessing mental capacity in DD patients is associated with certain challenges. These include psychopathological comorbidities, nutritional deficits, low education, previous head trauma, and cognitive deficits [23]. Moreover, the use of substances has an impact on an individual’s ability to make decisions. For example, amphetamine and heroin chronic use may lead to distinct patterns of cognitive impairment (e.g., in recognition memory, spatial working memory, planning, sequence generation, visual discrimination learning, and attentional set-shifting) that may be associated with dysfunction of different components of cortico-striatal circuitry [24,25]. Chronic cocaine use can be associated with dysfunction of the orbitofrontal cortex (OFC), the brain region involved in decision-making processes [26,27]. Chronic amphetamine users have shown deficits in attention, planning, decision-making, inhibitory control, and memory and learning domains [28,29]. This is also demonstrated in animal models [30]. Chronic MDMA use is specifically associated with decreased performance in declarative memory and additional deficits in working memory and executive functions [31]. Long-term cannabis users perform significantly less well on memory and attention tests, showing impaired learning, retention, and retrieval functions compared with shorter-term users and controls [32]. Alcohol use disorder (AUD) is associated with an increased risk of cognitive impairments, Alzheimer’s disease, and dementia, especially vascular dementia. AUD interacts with comorbidities, increasing the risk of cognitive impairment [33]. A reduced gray matter volume, especially in areas of the brain involved in decision-making, has been documented in individuals with AUD [34].

Data from the available literature show that cognitive deficits are detectable in about 50% of individuals with chronic SUDs [35,36,37], raising clinical, legal, and political issues in the management of DD patients [38]. These data highlight concerns about the ability of DD patients to understand and remember information (key components of a capacity assessment) and the acquisition of informed consent for both clinical and research purposes [13]. Despite this, our data suggest that the ability to make decisions related to health is not routinely assessed in this group.

A comparative study amongst 53 subjects (case group) with SUDs (alcohol, cannabis, cocaine) and 50 controls with other medical conditions (arterial hypertension, diabetes mellitus, and other minor medical pathologies) showed that the capacity to provide informed consent for scientific research was lower in the case group, with a significant reduction in the “understanding” and “evaluation” dimensions detected through a binary judgment of capacity/incapacity guided by the MacArthur Competence Assessment Tool for Clinical Research (MacCAT–CR) and a clinical interview [39]. Similarly, a study assessing the competence (i.e., decision-making capacity from a legal perspective) to provide informed consent to scientific research in a group of 77 subjects self-referred to an outpatient service for alcohol and drug use demonstrated that only 15% of the subjects provided consistent answers to a 14-item true/false quiz that assessed their comprehension of basic information provided in the consent form [40].

Despite the evident need for capacity assessment, our results indicate that they are frequently not undertaken in this population, and that even less commonly do they relate to the capacity to understand the implications of substance abuse. It is likely that the difficulty in assessing capacity caused by the aforementioned neuropsychiatric changes goes some way towards explaining this. The existing literature questions whether current capacity tools (such the MCA used in England and Wales) provide sufficient support for capacity assessment in the SUD clinical population. While generating a list of key principles—including psychiatric, physical, and cognitive examination—Nassif [13] suggested a flexible yet structured method using the MacArthur Competence Assessment Tool for Treatment (Mac-CAT-T) [41], though not specific to the SUD clinical population. They concluded that “*assessing the decisional capacity of a patient with a substance use disorder can be challenging. Primary or secondary conditions related to substance use can affect a patient’s decisional capacity on a temporary or permanent basis. A skilled psychiatric evaluation that includes a thorough cognitive examination and is complemented by legal or ethical consultation can help in making judicious decisions*”. Further work towards determining a robust method by which to assess capacity could facilitate improved rates of assessment.

We acknowledge certain limitations to our methodology. Namely, this was performed in a single center with only 696 admissions during the relevant period. Given that the analysis was retrospective, we were only able to include capacity assessments which were recorded in patient notes; it may be that some were performed but not documented. While we have identified a deficiency in rates of assessment, we were not able to collect data regarding outcomes (e.g., subsequent substance abuse). As such, we are not able to demonstrate the impact of assessments on clinical outcomes in this population. Unfortunately, logistic regression could not be performed on the sample of patients undergoing capacity assessments relating to substance use due to insufficient sample size.

## 5. Conclusions

Substance use in patients with mental health conditions is associated with worse outcomes and impaired decision-making [4].

Our findings indicate low rates of capacity assessment across acute psychiatric admissions (34.9%), with very few relating to capacity to understand the negative impact of substances (2.1%). The proportion of patients who lack capacity to make these decisions is likely to be significant. Lack of identification deprives patients without capacity of additional protective measures. Likewise, identifying patients who do have capacity is vital to facilitate shared decision-making. It is therefore vital that rates of assessment are improved. In the UK, this is currently performed as per the MCA (2005) [15]; however, formulation of a more structured method of assessment would likely facilitate improved completion.

Further research comparing outcomes between dual diagnosis patients who have a capacity assessment recorded versus those who do not would be beneficial.

## Figures and Tables

**Table 1:** Demographic profile of admissions based on completion of capacity assessment.

		If Capacity Was Assessed		
Variable	Total Admissions N = 696	NO N = 453 (65.1%)	YES N = 243 (34.9%)	Test Statistics
**Admission Age (years)**	43.0 (11.3)	42.5 (12.8)	43.9 (7.4)	t = −1.8, df = 690, *p* = 0.067
**Gender**				x^2^ = 55, df = 1, *p* < 0.001
Men	571 (82.3%)	335 (74.3%)	236 (97.1%)	
Women	123 (17.7%)	116 (25.7%)	7 (2.9%)	
**Ethnicity**				x^2^ = 63, df = 4, *p* < 0.001
White	270 (39.2%)	217 (48.8%)	53 (21.8%)	
Other	37 (5.4%)	25 (5.6%)	12 (4.9%)	
Mixed	52 (7.6%)	31 (7.0%)	21 (8.6%)	
Black	280 (40.7%)	136 (30.6%)	144 (59.3%)	
Asian	49 (7.1%)	36 (8.1%)	13 (5.3%)	
**Employment**				x^2^ = 143, df = 3, *p* < 0.001
Employed	42 (7.4%)	42 (12.3%)	0 (0.0%)	
Other	7 (1.2%)	7 (2.1%)	0 (0.0%)	
Sick pay	396 (69.4%)	172 (50.4%)	224 (97.4%)	
Unemployed	126 (22.1%)	120 (35.2%)	6 (2.6%)	
**Relationships status**				x^2^ = 5.5, df = 1, *p* = 0.019
Married or living with a partner	44 (6.5%)	36 (8.2%)	8 (3.3%)	
Single	636 (93.5%)	401 (91.8%)	235 (96.7%)	

**Table 2:** Clinical presentation of admissions with and without completed capacity assessments.

		Total Admissions	Capacity Assessment Completed		Test Statistics
			No	Yes	
		(N = 696)	(N = 453, 65.1%)	(N = 243, 34.9%)	
**Admission Ward Location**					x^2^ = 141, df = 1, *p* < 0.001
	PICU	403 (57.9%)	188 (41.5%)	215 (88.5%)	
	Acute Ward	293 (42.1%)	265 (58.5%)	28 (11.5%)	
**Primary diagnoses**					x^2^ = 61, df = 5, *p* < 0.001
	Mental and behavioural disorder due to substances	32 (4.7%)	17 (3.8%)	15 (6.2%)	
	Mood [affective] disorders	96 (14.0%)	90 (20.2%)	6 (2.5%)	
	Neurotic, stress-related, and somatoform	9 (1.3%)	9 (2.0%)	0 (0.0%)	
	Others	34 (4.9%)	30 (6.7%)	4 (1.7%)	
	Personality disorders	51 (7.4%)	32 (7.2%)	19 (7.9%)	
	Schizophrenia spectrum	466 (67.7%)	268 (60.1%)	198 (81.8%)	
**Secondary diagnoses**					x^2^ = 93, df = 14, *p* < 0.001
	Anorexia	2 (0.7%)	2 (1.4%)	0 (0.0%)	
	Anxiety disorder	6 (2.2%)	6 (4.1%)	0 (0.0%)	
	Autism	5 (1.9%)	5 (3.4%)	0 (0.0%)	
	Degenerative disease	5 (1.9%)	5 (3.4%)	0 (0.0%)	
	Mental and behavioural disorder due to substances	55 (20.4%)	37 (25.3%)	18 (14.6%)	
	Mood affective disorder	3 (1.1%)	3 (2.1%)	0 (0.0%)	
	Others	24 (8.9%)	12 (8.2%)	12 (9.8%)	
	Personality disorders	11 (4.1%)	11 (7.5%)	0 (0.0%)	
	Schizophrenia spectrum	29 (10.8%)	11 (7.5%)	18 (14.6%)	
**MHA**					x^2^ = 14, df = 1, *p* < 0.001
	No	124 (17.8%)	99 (21.9%)	25 (10.3%)	
	Yes	572 (82.2%)	354 (78.1%)	218 (89.7%)	

PICU: psychiatric intensive care unit; MHA: Mental Health Act.

## Data Availability

The original contributions presented in this study are included in the article. Further inquiries can be directed to the corresponding author.

## References

[1] NICE Guideline (NG58) Coexisting Severe Mental Illness and Substance Misuse: Community Health and Social Care Services. 30 November 2016. https://www.nice.org.uk/guidance/NG58.

[2] Marcó-García S., Ariyo K., Owen G.S., David A.S. (2024). Decision making capacity for treatment in psychiatric inpatients: A systematic review and meta-analysis. Psychol. Med..

[3] Owen G.S., David A.S., Richardson G., Szmukler G., Hayward P., Hotopf M. (2009). Mental capacity, diagnosis and insight in psychiatric in-patients: A cross-sectional study. Psychol. Med..

[4] Dixon L. (1999). Dual diagnosis of substance abuse in schizophrenia: Prevalence and impact on outcomes. Schizophr. Res..

[5] World Health Organization (2024). WHO Resources for Mental Health [Internet].

[6] University of Manchester (2017). The National Confidential Inquiry into Suicide and Homicide by People with Mental Illness.

[7] Safer Communities Directorate Homicide in Scotland 2015–1016: Statistics. 11 October 2016. https://www.gov.scot/publications/homicide-scotland-2015-16/pages/3/.

[8] Hoover D.B., Korthuis P.T., Waddell E.N., Foot C., Conway C., Crane H.M., Friedmann P.D., Go V.F., Nance R.M., Pho M.T. (2023). Recent Incarceration, Substance Use, Overdose, and Service Use Among People Who Use Drugs in Rural Communities. JAMA Netw. Open.

[9] Szerman N., Lopez-Castroman J., Arias F., Morant C., Babín F., Mesías B., Basurte I., Vega P., Baca-García E. (2012). Dual Diagnosis and Suicide Risk in a Spanish Outpatient Sample. Subst. Use Misuse.

[10] Lukasiewicz M., Blecha L., Falissard B., Neveu X., Benyamina A., Reynaud M., Gasquet I. (2009). Dual Diagnosis: Prevalence, Risk Factors, and Relationship with Suicide Risk in a Nationwide Sample of French Prisoners. Alcohol. Clin. Exp. Res..

[11] Schulte S., Holland M. (2008). Dual diagnosis in Manchester, UK: Practitioners’ estimates of prevalence rates in mental health and substance misuse services. Ment. Health Subst. Use.

[12] Lehman A.F., Myers C.P., Corty E., Thompson J.W. (1994). Prevalence and patterns of “dual diagnosis” among psychiatric inpatients. Compr. Psychiatry.

[13] Nassif W. (2019). Assessing decisional capacity in patients with substance use disorders. Curr. Psychiatr..

[14] Koffarnus M.N., Kaplan B.A. (2018). Clinical models of decision making in addiction. Pharmacol. Biochem. Behav..

[15] Parliament of the United Kingdom (2005). Mental Capacity Act 2005. 9 United Kingdom.

[16] Oireachtas (2015). Assisted Decision-Making (Capacity) Act 2015 [Internet]. No. 64 of 2015, Ireland. https://www.irishstatutebook.ie/eli/2015/act/64/enacted/en/html.

[17] Government of Singapore (2008). Mental Capacity Act 2008 (No. 22 of 2008) [Internet]. Singapore Statutes Online. https://sso.agc.gov.sg/Act/MCA2008.

[18] Okai D., Owen G., McGuire H., Singh S., Churchill R., Hotopf M. (2007). Mental capacity in psychiatric patients. Br. J. Psychiatry.

[19] Bradley K.A., Kivlahan D.R. (2014). Bringing patient-centered care to patients with alcohol use disorders. JAMA.

[20] Mental Capacity Act 2005 (2005). Crown Copyright. http://www.legislation.gov.uk/ukpga/2005/9/pdfs/ukpga_20050009_en.pdf.

[21] R Core Team (2022). A Language and Environment for Statistical Computing.

[22] Ariyo K., McWilliams A., David A.S., Owen G.S. (2021). Experiences of assessing mental capacity in England and Wales: A large-scale survey of professionals. Wellcome Open Res..

[23] McCrady B.S., Bux D.A. (1999). Ethical issues in informed consent with substance abusers. J. Consult. Clin. Psychol..

[24] Tolomeo S., Steele J.D., Ekhtiari H., Baldacchino A. (2021). Chronic heroin use disorder and the brain: Current evidence and future implications. Prog. Neuro-Psychopharmacol. Biol. Psychiatry.

[25] Ornstein T.J., Iddon J.L., Baldacchino A.M., Sahakian B.J., London M., Everitt B.J., Robbins T.W. (2000). Profiles of cognitive dysfunction in chronic amphetamine and heroin abusers. Neuropsychopharmacology.

[26] Lucantonio F., Stalnaker T., Shaham Y., Niv Y., Schoenbaum G. (2012). The impact of orbitofrontal dysfunction on cocaine addiction. Nat. Neurosci..

[27] Bolla K.I., Eldreth D.A., London E.D., Kiehl K.A., Mouratidis M., Contoreggi C., Matochik J.A., Kurian V., Cadet J.L., Kimes A.S. (2003). Orbitofrontal cortex dysfunction in abstinent cocaine 86 abusers performing a decision-making task. Neuroimage.

[28] Potvin S., Pelletier J., Grot S., Hébert C., Barr A.M., Lecomte T. (2018). Cognitive deficits in individuals with methamphetamine use disorder: A meta-analysis. Addict. Behav..

[29] Ersche K.D., Sahakian B.J. (2007). The neuropsychology of amphetamine and opiate dependence: Implications for treatment. Neuropsychol. Rev..

[30] Mizoguchi H., Yamada K. (2019). Methamphetamine use causes cognitive impairment and altered decision-making. Neurochem. Int..

[31] Wunderli M.D., Vonmoos M., Fürst M., Schädelin K., Kraemer T., Baumgartner M.R., Seifritz E., Quednow B.B. (2017). Discrete memory impairments in largely pure chronic users of MDMA. Eur. Neuropsychopharmacol..

[32] Solowij N., Stephens R.S., Roffman R.A., Babor T., Kadden R., Miller M., Christiansen K., McRee B., Vendetti J., Marijuana Treatment Project Research Group (2002). Cognitive functioning of long-term heavy cannabis users seeking treatment. JAMA.

[33] Wang G., Li D.Y., Vance D.E., Li W. (2023). Alcohol use disorder as a risk factor for cognitive impairment. J. Alzheimer’s Dis..

[34] Le Berre A.P., Rauchs G., La Joie R., Mézenge F., Boudehent C., Vabret F., Allain P., Eustache F., Pitel A.-L., Beaunieux H. (2014). Impaired decision-making and brain shrinkage in alcoholism. Eur. Psychiatry.

[35] Ramey T., Regier P.S. (2019). Cognitive impairment in substance use disorders. CNS Spectr..

[36] Bruijnen C.J.W.H., Dijkstra B.A.G., Walvoort S.J.W., Markus W., VanDerNagel J.E.L., Kessels R.P.C., De Jong C.A.J. (2019). Prevalence of cognitive impairment in patients with substance use disorder. Drug Alcohol. Rev..

[37] Bates M.E., Convit A., Calev A. (1999). Neuropsychology and neuroimaging of alcohol and illicit drug abuse. Assessment of Neuropsychological Functions in Psychiatric Disorders.

[38] Jeste D.V., Saks E. (2006). Decisional capacity in mental illness and substance use disorders: Empirical database and policy implications. Behav. Sci. Law..

[39] Morán-Sánchez I., Luna A., Sánchez-Muñoz M., Aguilera-Alcaraz B., Pérez-Cárceles M.D. (2016). Decision-making capacity for research participation among addicted people: A cross-sectional study. BMC Med. Ethics.

[40] Kiluk B.D., Nich C., Carroll K.M. (2010). Neurocognitive indicators predict results of an informed-consent quiz among substance-dependent treatment seekers entering a randomized clinical trial. J. Stud. Alcohol. Drugs.

[41] Grisso T., Appelbaum P.S., Grisso T., Appelbaum P.S. (1998). Chapter 6: Using the MacArthur competence assessment tool—Treatment. Assessing Competence to Consent to Treatment: A Guide for Physicians and Other Health Professionals.

